# Effects of cAMP and CFTR modulation on apical fluid pH in human airway Calu‐3 cells

**DOI:** 10.14814/phy2.70747

**Published:** 2026-02-03

**Authors:** Jenny P. Nguyen, Nadia Milad, Jeremy A. Hirota

**Affiliations:** ^1^ Division of Respirology, Department of Medicine, Firestone Institute for Respiratory Health McMaster University Hamilton Ontario Canada; ^2^ McMaster Immunology Research Centre McMaster University Hamilton Ontario Canada; ^3^ Division of Respiratory Medicine, Department of Medicine University of British Columbia Vancouver British Columbia Canada; ^4^ Department of Biology University of Waterloo Waterloo Ontario Canada

**Keywords:** apical fluid pH regulation, cAMP signaling, CFTR modulation, human airway epithelial cells

## Abstract

The airway epithelium serves as the first line of defense against inhaled insults present in the external environment by acting as a physical barrier and through host defense mechanisms. Proper maintenance of these host defense mechanisms relies on the regulation of airway surface liquid (ASL) composition and properties, a process that is tightly controlled by various ion transporters, including the cystic fibrosis transmembrane conductance regulator (CFTR) protein. With evidence suggesting dysfunctional CFTR‐mediated bicarbonate secretion leads to airway acidification, resulting in impaired host defenses, there is increased interest in improving ASL pH. The aim of our study was to determine whether pharmacological interventions, via cAMP and CFTR modulators, lead to an increase in pH. Human airway epithelial (Calu‐3) cells were exposed to various combinations of cAMP and CFTR modulating agents to assess their effectiveness at elevating apical base secretions (apical fluid) pH. Our results show that pharmacological interventions with cAMP elevating agents and CFTR modulator VX‐770 led to significant increases in pH, with combinations leading to greater increases compared to single drug interventions. Our study suggests that cAMP and CFTR modulation has potential as a therapeutic strategy for elevating ASL pH and may be beneficial for respiratory diseases with ASL abnormalities.

## 1 INTRODUCTION

The airway epithelium is the first line of defense against harmful pathogens and inhaled particles present in the external environment by acting as a physical barrier and by initiating various host defense mechanisms, including mucociliary clearance (MCC) and antimicrobial activity (Hiemstra et al., 2015; Knowles & Boucher, 2002). MCC is an innate host defense mechanism that protects the lungs by capturing pathogens and particulates in the mucus and expelling them out of the airways (Haq et al., 2016; Hiemstra et al., 2015; Knowles & Boucher, 2002). It is a highly coordinated process that is dependent on several different components, including proper ciliary function and airway surface liquid (ASL) (Knowles & Boucher, 2002; Zajac et al., 2021). The ASL, which is comprised of a periciliary layer and a mucus layer, contributes to proper airway hydration. In addition, the ASL also contains antimicrobial agents, thus contributing to antimicrobial host defenses (Haq et al., 2016; Hiemstra et al., 2015; Knowles & Boucher, 2002; Zajac et al., 2021). The ASL composition, as well as the volume and pH, is highly regulated by various ion transporters, including the cystic fibrosis transmembrane conductance regulator (CFTR) protein (Alaiwa et al., 2014; Berkebile & McCray, 2014; Haq et al., 2016; Hiemstra et al., 2015; Jayaraman, Joo, et al., 2001; Pezzulo et al., 2012; Tang et al., 2016; Zajac et al., 2021). Due to its critical role for normal airway function, dysregulation of ASL is associated with various diseases, including cystic fibrosis (CF) (Haq et al., 2016; Zajac et al., 2021).

CF is a recessive genetic disease caused by mutations in both alleles of the CFTR gene (Riordan et al., 1989). CFTR is a phosphorylation‐dependent ion channel that conducts chloride and bicarbonate ions across the epithelia, contributing to epithelial fluid transport in the lungs (Anderson et al., 1991; Bear et al., 1992; Shan et al., 2012; Smith & Welsh, 1992). Due to CFTR dysfunction, individuals with CF experience several complications, including impaired MCC, reduced antimicrobial activity, abnormal inflammatory responses, persistent airway infections, and airway obstruction (Alaiwa et al., 2014; Jayaraman, Joo, et al., 2001; Pezzulo et al., 2012; Simonin et al., 2019; Tang et al., 2016). Collectively, these consequences can result in the loss of pulmonary function, the major cause of morbidity and mortality in CF. The pathogenesis of CF lung disease has been suggested to be initiated by defective airway host defenses, thus predisposing CF individuals to persistent airway infections and increased inflammation (Alaiwa et al., 2014; Haq et al., 2016; Hiemstra et al., 2015; Pezzulo et al., 2012).

Currently, there exist pharmacological interventions for the treatment of CFTR dysfunction for select CFTR mutations called CFTR modulators, which are small molecules designed to correct and improve the function of CFTR (Keating et al., 2018; Middleton et al., 2019; Taylor‐Cousar et al., 2017; Van Goor et al., 2009, 2011). Presently, there are five CFTR modulator treatments approved by the Federal Drug Administration, Kalydeco (ivacaftor; VX‐770), Orkambi (lumacaftor/ivacaftor; VX‐809/VX‐770), Symdeko (tezacaftor/ivacaftor; VX‐661/VX‐770), Trikafta (elexacaftor/tezacaftor/ivacaftor; VX‐445/VX‐661/VX‐770), and Alyftrek (vanzacaftor/tezacaftor/deutivacaftor; VX‐121/VX‐661/VX‐561) (*KALYDECO (ivacaftor) package insert*, 2025; *ORKAMBI (lumacaftor/ivacaftor) package insert*, 2025; *SYMDEKO (tezacaftor/ivacaftor) package insert*, 2025; *TRIKAFTA (elexacaftor/tezacaftor/ivacaftor) package insert*, 2025; *ALYFTREK (vanzacaftor/tezacaftor/deutivacaftor) Package Insert*, 2025). The use of CFTR modulators has been shown to be beneficial for CF management via improvement in lung function and reduction in pulmonary exacerbations (Accurso et al., 2010; Boyle et al., 2014; Hoppe et al., 2025; Keating et al., 2025; Ramsey et al., 2011; Uluer et al., 2023; Wainwright et al., 2015).

In addition to existing CFTR modulators, there have been several investigations into other molecules that can impact CFTR function, including drugs that can modulate cAMP metabolism. Elevated intracellular cAMP signaling activates protein kinase A, which phosphorylates CFTR and promotes channel opening (Cheng et al., 1991; Picciotto et al., 1992; Winter & Welsh, 1997; Zhang et al., 2018). Intracellular cAMP levels are regulated by various mechanisms, including G protein‐coupled receptors, adenylyl cyclases, cAMP‐efflux transporters, and phosphodiesterases (Ahmadi et al., 2017; Cobb et al., 2003; Conner et al., 2013; Lambert et al., 2014; Li et al., 2007). Previously, we have demonstrated that pharmacological inhibition of cAMP‐efflux transporter ATP binding cassette transporter C4 (ABCC4), in combination with CFTR potentiator VX‐770, increases CFTR function beyond VX‐770 alone in G551D‐CFTR subjects (Ahmadi et al., 2017). Additionally, we have also demonstrated that cAMP modulation, via inhibition of ABCC4 and phosphodiesterase‐4 (PDE‐4), in combination with VX‐770, is able to increase sensitivity and CFTR activity in Calu‐3 cells, when compared to VX‐770 treatment alone (Nguyen, Bianca, et al., 2021). Furthermore, we previously showed that pharmacological intervention with VX‐770 was able to rescue acquired CFTR dysfunction caused by diesel exhaust particles (Nguyen, Huff, et al., 2021). Altogether, these studies suggest that combining cAMP modulation with CFTR modulators can be used to improve CFTR function to potentially increase ASL pH.

In this study, we hypothesized that the potentiation of CFTR function in human airway epithelial cells, via cAMP modulation and CFTR modulators, will lead to an increase in apical base secretions (apical fluid) pH. To investigate the effect of cAMP and CFTR modulation on pH, human airway epithelial Calu‐3 cells were exposed to various pharmacological interventions. We demonstrate that pharmacological interventions with combinations of cAMP and CFTR modulating agents led to increases in apical fluid pH but cannot be solely attributed to CFTR. These results suggest the involvement of additional ion transporters and mechanisms in maintaining airway surface liquid homeostasis, potentially compensating for impaired CFTR function. Collectively, our results provide supporting evidence of cAMP and CFTR modulation as a therapeutic strategy for elevating pH, in line with previous studies using clinically relevant airway epithelial models while emphasizing the need for further research to understand its downstream effects and broader implications in ASL homeostasis.

## 2 MATERIALS AND METHODS

### 2.1 Reagents

All pharmacological intervention compounds were purchased from Cayman Chemical, excluding Ivacaftor, CFTR Inh‐172, and GlyH‐101, which were purchased from Selleck Chemicals. For cAMP modulation, cAMP elevating agent Forskolin (FSK; 66575‐29‐9) and Isoproterenol (ISO; 51‐30‐9), ABCC4 inhibitor MK‐571 (115104‐28‐4), and PDE‐4 inhibitor Roflumilast (RF; 162401‐32‐3) were used. For CFTR modulation, CFTR potentiator Ivacaftor (VX‐770; 873054‐44‐5) and CFTR inhibitors CFTR Inh‐172 (CFTRinh‐172; 307510‐92‐5) and GlyH‐101 (328541‐79‐3) were used. Concentrations greater than 10 μM were not used for CFTR inhibition as both inhibitors are known to exhibit off‐target effects on other ion channels and transporters at elevated doses (Ma et al., 2002; Melis et al., 2014). All pharmacological intervention compounds were dissolved in DMSO (67‐68‐5).

### 2.2 Human airway epithelial cell culture

The human airway epithelial cell line Calu‐3 (male, age 25) (ATCC, HTB‐55), derived from lung adenocarcinoma tissue, was cultured in Alpha‐Minimum Essential medium (α‐MEM) (Corning, 10‐022‐CV) supplemented with 10% fetal bovine serum (VWR, 080‐450), 1% HEPES (Corning, 25‐060‐CI), and 1% penicillin–streptomycin (VWR, 97063‐708) at 37°C and 5% CO_2_ for both submerged and air‐liquid interface (ALI) culture conditions. The medium was changed three times per week (every Monday, Wednesday, and Friday). Calu‐3 cells were grown until they reached 50%–70% confluency and were subsequently passaged and plated onto 6.5 mm Transwell with 0.4 μm Pore Inserts (Corning, 3470). Medium changes to both the apical (top) and basolateral (bottom) compartment were performed until 100% confluency was reached. Calu‐3 cells were then grown at ALI for at least 21 days post‐air lift prior to apical fluid pH experiments.

### 2.3 Transepithelial electrical resistance (TEER) measurements

Electrical resistance measurements were performed using the Millicell ERS‐2 Epithelial Volt‐Ohm Meter (Millipore, MERS00002) after washing the apical surface of the cell layer with 200 μL of warmed PBS (Corning, 21‐040‐CV) (10 min) and feeding the basolateral compartment with complete α‐MEM. TEER values were subsequently calculated and used as a method for quality control to confirm the integrity and permeability of the cell layer on the Transwell insert. For all experiments, the averaged TEER values for the Transwell inserts were typically ≥200 Ω*cm^2^.

### 2.4 Apical base secretions “apical fluid” pH measurements

Calu‐3 cells were washed with warmed PBS prior to experimental conditions and TEER measurements were performed 1 h prior to stimulation with pharmacological interventions. For apical fluid pH experiments, the apical compartment was loaded with 100 μL of HCO_3_^−^ and K^+^ − free saline Ringer's Solution (135 mM NaCl, 1.2 mM CaCl_2_, 1.2 mM MgCl_2_, 2.5 mM Na_2_HPO_4_; pH 6), at physiological ionic strength and with low phosphate content to minimize buffering. The basolateral compartment was replaced with HEPES‐free α‐MEM supplemented with 10% fetal bovine serum and 1% penicillin–streptomycin. Pharmacological interventions were administered to the basolateral compartment for 3 h at 37°C, unless otherwise stated. After 3 h, plates were left at RT for 30 min prior to collection of the fluid in the apical compartment into microcentrifuge tubes. Tubes were spun down at 7500 rpm for 10 min to remove any cells and mucus, then subsequently transferred to a new set of tubes. After collection of the cell‐ and mucus‐free apical base secretions (apical fluid), pH was measured using the commercially available Orion PerpHecT ROSS Combination pH Micro Electrode (Thermo Scientific, 8220BNWP). Prior to all pH measurements, the pH microelectrode was calibrated using the appropriate buffers and was rinsed with ddH_2_O between measurements. Following collection of the apical fluid, if cells were intended for subsequent experiments, the basolateral compartment was replaced with complete α‐MEM and transferred into the incubator at 37°C and 5% CO_2_ for future use.

### 2.5 Statistical analysis

For apical fluid pH experiments, a single pH measurement was taken per Transwell insert and measured pH values were averaged across all biological replicates (*n* = 3–7). SD was calculated using data from all biological replicates (*n* = 3–7), and statistical analysis was performed using one‐way or two‐way ANOVA with subsequent multiple comparisons. All analyses were performed using GraphPad Prism 10.

## 3 RESULTS

### 3.1 cAMP elevating agents increase apical fluid pH

In our previous study, we demonstrated the ability of cAMP elevating agents FSK and ISO to induce CFTR activity in human airway epithelial Calu‐3 cells (Nguyen, Bianca, et al., 2021). Furthermore, we also demonstrated the capacity of FSK to elicit a rise in apical fluid pH (Dabaghi et al., 2021). FSK and ISO induce CFTR activity indirectly by increasing intracellular cAMP via distinct mechanisms. FSK directly activates adenylyl cyclase in a receptor‐independent manner, bypassing membrane receptors and potentially stimulating multiple adenylyl cyclase isoforms throughout the cell (Seamon et al., 1981). This broad activation can result in a more global rise in cAMP. In contrast, ISO is a β‐adrenergic receptor agonist that elevates cAMP through receptor‐dependent G protein‐mediated signaling, restricting cAMP generation to receptor‐associated adenylyl cyclases (Naren et al., 2003; Vijftigschild et al., 2016).

**FIGURE 1:**
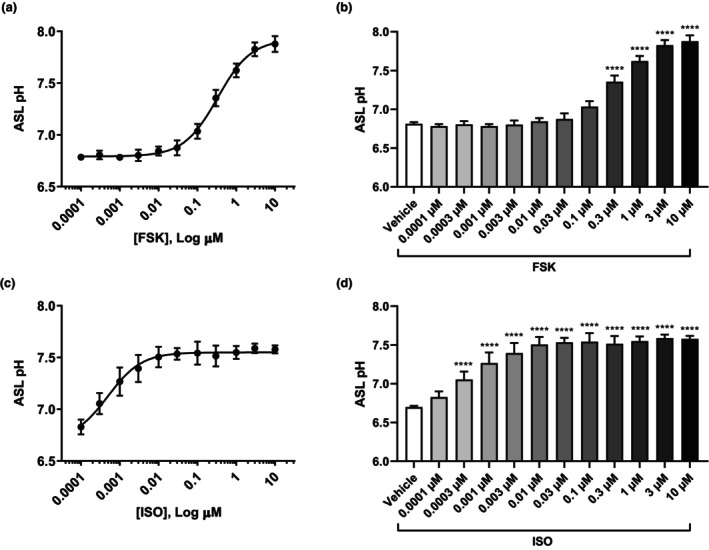
Concentration‐response analysis of cAMP elevating agents on apical fluid pH. Human airway epithelial (Calu‐3) cells were exposed to cAMP elevating agent (a and b) forskolin and (c and d) isoproterenol in the basolateral (bottom) compartment for 3 h at various concentrations. (a and c) A dose–response curve for measured apical fluid pH is depicted and (b and d) comparisons between treatment groups were performed. Data presented as means ± SD (*n* = 4–5, FSK; *n* = 5, ISO). A one‐way ANOVA with subsequent multiple comparisons was used for statistical analysis. ****p ≤ 0.0001.

To validate whether enhanced CFTR activity corresponds to increases in apical fluid pH, Calu‐3 cells grown under ALI conditions were exposed to FSK or ISO for 3 h in the basolateral compartment (Figure 1). Cells treated with FSK showed a dose‐dependent increase in apical fluid pH (Figure 1a) and comparisons between treatment groups confirmed these increases to be significant (Figure 1b; ****p ≤ 0.0001). Similarly, ISO treatment also resulted in a dose‐dependent increase in apical fluid pH (Figure 1c), with significant differences between groups (Figure 1d; ****p ≤ 0.0001). These findings align with previous studies demonstrating that the introduction of cAMP elevating agents results in a rise in pH (Coakley et al., 2003; Dabaghi et al., 2021; Tang et al., 2016). Altogether, these findings indicate that cAMP elevating agents FSK and ISO not only induce CFTR activity but also increase apical fluid pH.

### 3.2 pH is unaltered in the absence of cells

**FIGURE 2:**
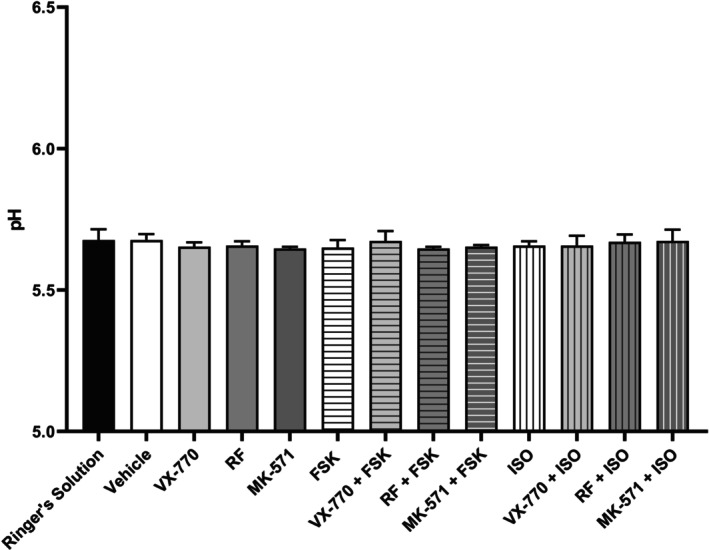
Control experiment in Ringer's solution to validate drug‐induced pH changes in the absence of cells. Various combinations of cAMP elevating agents forskolin (0.1 μM) and isoproterenol (0.01 μM), CFTR potentiator VX‐770 (1 μM), PDE‐4 inhibitor roflumilast (1 μM), and ABCC4 inhibitor MK‐571 (10 μM) were administered to the basolateral (bottom) compartment for 3 h to evaluate the change in pH in the absence of human airway epithelial (Calu‐3) cells. Data presented as means ± SD (*n* = 3). A one‐way ANOVA with subsequent multiple comparisons was used for statistical analysis.

To confirm whether the observed changes in apical fluid pH are due to cellular responses, rather than the direct addition of the drugs, we tested various pharmacological interventions in the absence of Calu‐3 cells (Figure 2). No significant differences in pH were observed in response to FSK, ISO, CFTR potentiator VX‐770, PDE‐4 inhibitor RF, ABCC4 inhibitor MK‐571, or combinations of these drugs. This suggests that the previously observed increases in apical fluid pH are likely attributed to cellular responses to the pharmacological interventions rather than the drug compounds themselves. It is worth noting that although Ringer's solution was prepared to an initial pH of 6, the measured initial pH in these experiments was ~5.6, likely reflecting its limited buffer capacity, residual bicarbonate, and potential effects of CO_2_ equilibration or temperature. While Ringer's solution was intended to be HCO_3_^−^‐free, residual bicarbonate may remain, potentially influencing subsequent apical fluid pH measurements, especially when the solution is exposed to air or once it is removed from the 5% CO_2_ incubator.

### 3.3 Impact of pharmacological interventions on apical fluid pH

**FIGURE 3:**
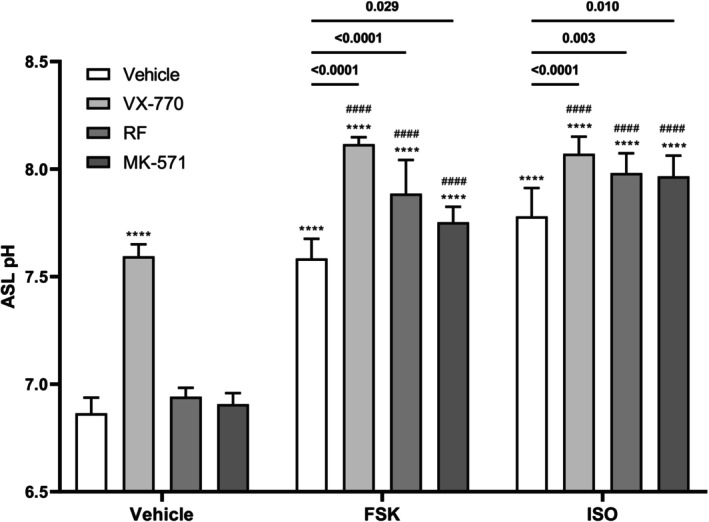
Effect of various pharmacological interventions on apical fluid pH. Human airway epithelial (Calu‐3) cells were exposed to various combinations of cAMP elevating agents forskolin (0.1 μM) and isoproterenol (0.01 μM), CFTR potentiator VX‐770 (1 μM), PDE‐4 inhibitor roflumilast (1 μM), and ABCC4 inhibitor MK‐571 (10 μM) to the basolateral (bottom) compartment for 3 h. Measured apical fluid pH is depicted and comparisons between treatment groups were performed. Data presented as means ± SD (*n* = 7). A two‐way ANOVA with subsequent multiple comparisons was used for statistical analysis. ****p ≤ 0.0001; ^####^ *p* ≤ 0.0001.

Having established that the observed changes in pH are linked to cellular responses, we next investigated the effect of various pharmacological interventions on apical fluid pH (Figure 3). Notably, the administration of VX‐770, FSK, and ISO, individually and in combination, resulted in significant increases in apical fluid pH (Figure 3; ****p ≤ 0.0001). This aligns with previous studies demonstrating that CFTR modulators can influence ASL properties (Chang et al., 2015; Ludovico et al., 2022; Van Goor et al., 2009). In contrast, the use of RF and MK‐571 alone did not lead to increases in apical fluid pH (Figure 3). However, when RF and MK‐571 were used in combination with a cAMP elevating agent, this led to a rise in apical fluid pH (Figure 3; ****p ≤ 0.0001). Furthermore, drug combinations led to greater increases in pH compared to their individual use (Figure 3; *p* = 0.029, *p* = 0.003, *p* = 0.010, *p* ≤ 0.0001, ****p ≤ 0.0001, ^####^ *p* ≤ 0.0001). Importantly, combining VX‐770 with cAMP elevating agents produced significantly greater increases in apical fluid pH than VX‐770 alone (Figure 3; ^####^ *p* ≤ 0.0001), highlighting cAMP and CFTR modulation as a potential therapeutic strategy for CFTR potentiation and ASL pH elevation. Overall, these findings suggest that combinational therapies are better at elevating apical fluid pH than single therapies, emphasizing the potential of cAMP and CFTR modulation as a therapeutic approach.

### 3.4 CFTR inhibition does not reduce apical fluid pH

Following our observations that pharmacological interventions modulating cAMP and CFTR, which have been shown to induce CFTR activity, can elevate apical fluid pH, we next explored whether apical or basolateral administration of CFTR inhibitors would reduce apical fluid pH by blocking CFTR‐mediated bicarbonate secretion.

**FIGURE 4:**
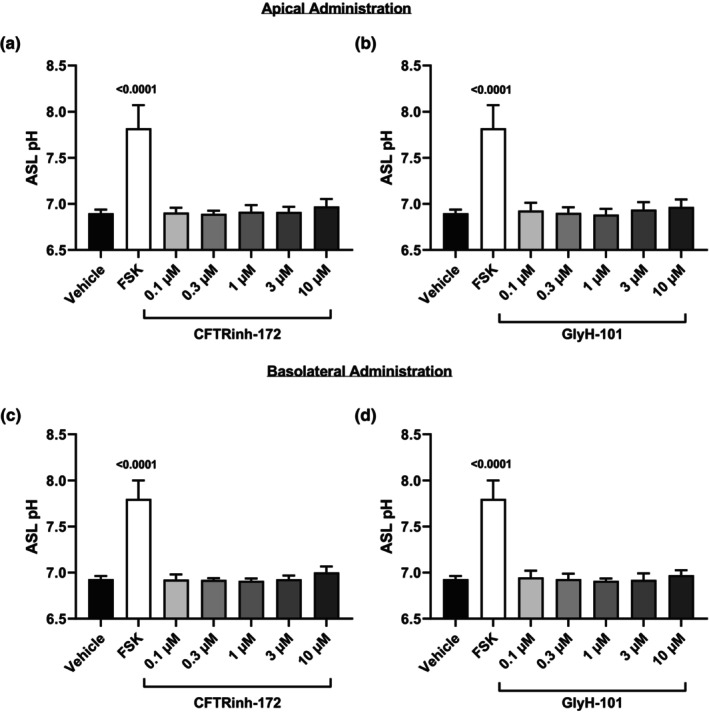
CFTR inhibitors administered in the apical or basolateral compartment do not impact apical fluid pH. Human airway epithelial (Calu‐3) cells were exposed to CFTR inhibitors (a and c) CFTRinh‐172 and (b and d) GlyH‐101 in the apical (a and b, top) or basolateral (c and d, bottom) compartment for 3 h at various concentrations. cAMP elevating agent forskolin (0.1 μM) was used as a positive control and comparisons between treatment groups were performed. Data presented as means ± SD (*n* = 5). A one‐way ANOVA with subsequent multiple comparisons was used for statistical analysis.

Calu‐3 cells were exposed to CFTR inhibitors CFTRinh‐172 and GlyH‐101 in the apical since CFTR is localized to the apical membrane or basolateral compartment for 3 h at various concentrations (up to 10 μM) (Figure 4) (Crawford et al., 1991; Denning et al., 1992). Apical administration of CFTRinh‐172 and GlyH‐101 (Figure 4a,b) did not lead to a reduction in apical fluid pH. Similarly, basolateral administration of CFTRinh‐172 and GlyH‐101 failed to reduce apical fluid pH (Figure 4c,d), suggesting that CFTR activity may be minimal at baseline and only contributes to pH regulation under stimulated conditions.

**FIGURE 5:**
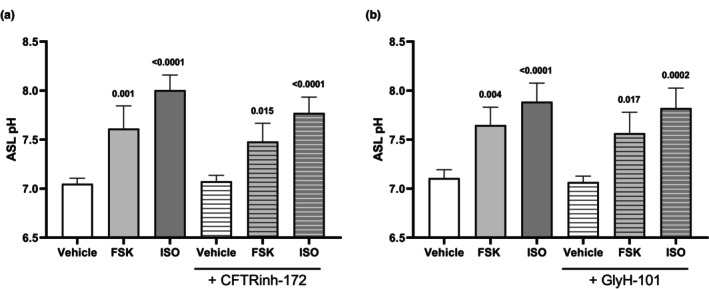
Effect of cAMP elevating agents FSK and ISO post‐CFTR inhibition on apical fluid pH. Human airway epithelial (Calu‐3) cells were pre‐treated with CFTR inhibitors (a) CFTRinh‐172 (10 μM) and (b) GlyH‐101 (10 μM) to the apical (top) compartment for 30 min prior to treatment with cAMP elevating agents forskolin (0.1 μM) and isoproterenol (0.01 μM) to the basolateral (bottom) compartment for 3 h. Measured apical fluid pH is depicted and comparisons between treatment groups were performed. Data presented as means ± SD (*n* = 4). A one‐way ANOVA with subsequent multiple comparisons was used for statistical analysis.

Considering that neither apical nor basolateral administration of CFTR inhibitors reduced apical fluid pH at baseline conditions, we next investigated whether pre‐treatment with CFTR inhibitors would prevent the observed increases in apical fluid pH induced by pharmacological interventions. Although pre‐treating Calu‐3 cells with CFTR inhibitors for 30 min to the apical compartment resulted in a reduced rise in apical fluid pH induced by cAMP elevating agents FSK and ISO (Figure 5), it was not of significance. Neither CFTRinh‐172 nor GlyH‐101 prevented increases in pH in response to cAMP elevating agents, suggesting that apical fluid pH changes by indirect CFTR activation are not impacted by CFTR inhibition. RF and MK‐571 were also tested, with no changes in apical fluid pH observed (Figure S1).

**FIGURE 6:**
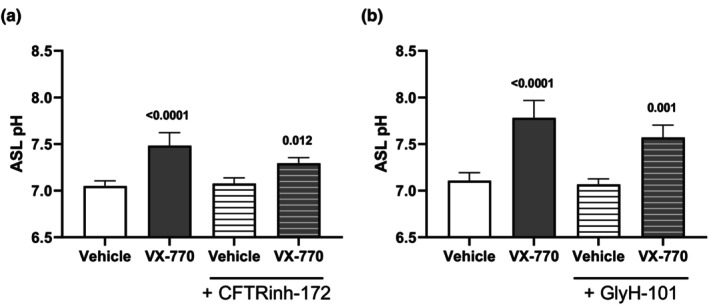
Effect of CFTR potentiator VX‐770 post‐CFTR inhibition on apical fluid pH. Human airway epithelial (Calu‐3) cells were pre‐treated with CFTR inhibitors (a) CFTRinh‐172 (10 μM) and (b) GlyH‐101 (10 μM) to the apical (top) compartment for 30 min prior to treatment with CFTR potentiator VX‐770 (1 μM) to the basolateral (bottom) compartment for 3 h. Measured apical fluid pH is depicted and comparisons between treatment groups were performed. Data presented as means ± SD (*n* = 4). A one‐way ANOVA with subsequent multiple comparisons was used for statistical analysis.

We next investigated the effect of pre‐treatment with CFTR inhibitors on VX‐770, a direct activator of CFTR (Figure 6). While pre‐treatment with CFTR inhibitors reduced the capacity of VX‐770 to elevate apical fluid pH, this reduction was not significant. Collectively, these findings suggest that while pharmacological interventions modulating cAMP or CFTR elevate apical fluid pH, increases in apical fluid pH cannot be solely attributed to CFTR, suggesting the involvement of alternative ion channels or transporters which may compensate for CFTR.

## 4 DISCUSSION

The airway epithelium plays a crucial role in protecting us against inhaled pathogens and particulates, relying on effective host defense mechanisms such as MCC and antimicrobial activity. These host defense mechanisms require proper regulation of ASL composition and properties. CFTR, the protein implicated in CF, plays a key role in ASL regulation, with CFTR dysfunction being associated with airway acidification and impaired host defenses. In the present study, we investigate the effect of pharmacological interventions, specifically cAMP and CFTR modulators, on pH. Using Calu‐3 cells, we demonstrate that cAMP elevating agents FSK and ISO, along with CFTR potentiator VX‐770, lead to significant increases in apical fluid pH. These results are consistent with our previous study, which showed the ability of these compounds to induce CFTR activity in Calu‐3 cells (Nguyen, Bianca, et al., 2021). Additionally, cAMP elevating agents, when used in combination with VX‐770, produced greater increases in apical fluid pH, suggesting a potential additive effect. These findings prompt further exploration into whether pharmacological interventions that enhance CFTR activity and elevate ASL pH can improve downstream consequences.

The addition of both FSK and ISO to Calu‐3 cells resulted in a dose‐dependent increase in apical fluid pH. The differences observed in their ability to elevate apical fluid pH may be due to their mechanistic differences, impacting the number of adenylyl cyclase isoforms being activated and intracellular cAMP levels. Importantly, we confirmed that alterations in pH do not occur in the absence of cells. These findings are consistent with our previous demonstration that cAMP elevating agents can potentiate CFTR activity and elevate apical fluid pH, supporting the notion that increases in CFTR activity may translate to elevations in ASL pH (Dabaghi et al., 2021; Nguyen, Bianca, et al., 2021). Our results align with other studies demonstrating a rise in ASL pH in response to cAMP elevation but contradict previous studies that indicated no significant impact of cAMP elevation on ASL pH (Coakley et al., 2003; Jayaraman, Song, & Verkman, 2001; Simonin et al., 2019; Tang et al., 2016). In preclinical models, treatment of wild‐type pig tracheas and CF ferret tracheas with FSK and low‐dose cholinergic agonist carbachol led to synergistically increased fluid secretion and enhanced mucociliary clearance velocity via increased anion secretion and decreased sodium absorption, though the impact on pH was not assessed in this study (Joo et al., 2021).

To further explore this, we next investigated the impact of pharmacological interventions, targeting cAMP and CFTR, on apical fluid pH. VX‐770, FSK, and ISO individually resulted in significant increases in apical fluid pH. In contrast, individual administration of PDE‐4 inhibitor RF or ABCC4 inhibitor MK‐571 did not elevate apical fluid pH. These findings align with our previous studies that assessed their ability to potentiate CFTR (Nguyen, Bianca, et al., 2021; Nguyen, Huff, et al., 2021). However, when VX‐770, RF, or MK‐571 was administered in combination with either cAMP‐elevating agent FSK or ISO, we observed significant increases in apical fluid pH. Moreover, these increases were more substantial than individual interventions, suggesting additive effects with drug combinations. Our findings are supported by previous demonstrations of these compounds influencing ASL properties (Coakley et al., 2003; Tang et al., 2016; Tyrrell et al., 2015). Although one study following newborns with CF starting VX‐770 treatment found that there was no effect on ASL pH by 6 months of age, they found that reductions in sweat Cl^−^ concentrations correlated with increases in ASL pH and with improvements in lung function (Abou Alaiwa et al., 2018). Altogether, these results highlight the potential of these interventions as a combinatorial therapeutic strategy for potentiating CFTR and elevating ASL pH.

Since pharmacological interventions using cAMP and CFTR modulators elevated apical fluid pH and dysfunctional CFTR has been linked to airway acidification, we next investigated whether CFTR inhibition would reduce apical fluid pH (Coakley et al., 2003; Pezzulo et al., 2012; Shah et al., 2016). We hypothesized that CFTR inhibition would lower apical fluid pH by limiting bicarbonate secretion; however, neither apical nor basolateral administration of CFTRinh‐172 or GlyH‐101 under resting conditions reduced pH. Furthermore, pre‐treatment with CFTR inhibitors did not block the apical fluid pH increases induced by cAMP and CFTR modulators. These results suggest that while CFTR contributes to pH regulation, other ion channels and transporters likely compensate for impaired CFTR function to maintain pH (Haq et al., 2016). Notably, VX‐770 still elevated apical fluid pH after pre‐treatment with CFTR inhibitors. VX‐770, a selective CFTR potentiator, binds directly to CFTR and enhances gating by increasing channel open probability and stabilizing the open conformation (Eckford et al., 2012; Jih & Hwang, 2013; Liu et al., 2019). Whereas CFTRinh‐172 stabilizes the closed channel, affecting gating without occluding the pore, GlyH‐101 inhibits CFTR by pore occlusion (Kopeikin et al., 2010; Ma et al., 2002; Muanprasat et al., 2004; Norimatsu et al., 2012; Verkman et al., 2013; Young et al., 2024). Although both are potent CFTR inhibitors, they act at distinct sites and through different mechanisms, potentially allowing VX‐770 to partially counteract their inhibitory effects, which may explain why VX‐770 elevated apical fluid pH despite pre‐treatment with CFTR inhibitors (Gao et al., 2024; Liu et al., 2024). To minimize off‐target effects, inhibitor concentrations were limited to 10 μM, as higher doses can affect other channels. CFTRinh‐172 begins to have off‐target effects at concentrations greater than 5 μM, while GlyH‐101 has off‐target effects at all concentrations used to inhibit CFTR, which may further explain the observed pH changes (Melis et al., 2014).

A limitation of this study is the use of Calu‐3 cells rather than primary airway epithelial cells from healthy donors and CF subjects. Incorporating primary cultures and CFTR knockout cell lines in future experiments will enhance the physiological relevance and general applicability of these findings, along with clarifying CFTR‐specific responses. Another limitation is the use of “nominally” HCO_3_^−^ and K^+^‐free saline Ringer's Solution, which may contain residual bicarbonate, influencing the measured pH. Future studies should use a CO_2_‐free incubator to confirm whether the observed effects on pH are bicarbonate‐dependent and to characterize underlying mechanisms. The K^+^‐free solution may also inhibit H^+^/K^+^‐ATPase (ATP12A), an apical proton pump that contributes to ASL pH regulation. Additionally, using a KCl‐based pH electrode in this solution could introduce measurement artifacts. Finally, future work should also examine how cAMP and CFTR modulation influence ASL pH may impact host defenses.

ASL pH regulation is mediated by various ion channels and transporters, including ATP12A, pendrin (SLC26A4) – an apical chloride/bicarbonate exchanger, TMEM16A – a calcium‐activated chloride channel, and ENaC – an epithelial sodium channel (Delpiano et al., 2023; Haggie et al., 2016; Kim et al., 2019; Nguyen & Hirota, 2019; Scudieri et al., 2018). While pendrin plays an important role in regulating ASL pH, it has been suggested that most bicarbonate secretion in Calu‐3 cells is through CFTR (Huang et al., 2018). Another important consideration is inflammation, which influences ASL pH by modifying various mechanisms (Guidone et al., 2022; Rehman et al., 2021; Rehman & Welsh, 2023; Zajac et al., 2023). Studies have shown that the presence of inflammatory cytokines, such as TNF‐ɑ and IL‐17 – two cytokines elevated in CF individuals – is able to increase ASL pH (Rehman et al., 2021; Zajac et al., 2023). This pH elevation is further enhanced with the administration of CFTR modulators (Rehman et al., 2021; Zajac et al., 2023). Altogether, the regulation of ASL pH involves a complex interplay of various ion channels and transporters, which can be further influenced by inflammation, warranting consideration in future studies.

In this study, we demonstrate the potential of combinatorial therapies targeting cAMP and CFTR for improving pH. Our findings suggest the effectiveness of targeting multiple pathways, direct and indirect CFTR activation, as drug combinations led to greater increases in apical fluid pH compared to individual interventions. However, it is important to note that the elevations in apical fluid pH observed cannot be solely attributed to CFTR, thus further investigation into other ion channels and transporters involved in ASL regulation, along with the influence of inflammatory cytokines, should be pursued to better understand the underlying mechanisms involved. Additional and future studies exploring the effect of these pharmacological interventions on host defense mechanisms, such as antimicrobial activity and MCC, using primary cultures and CFTR knockout cell lines, and in the absence of bicarbonate should be performed. Altogether, this study supports continued investigation and development of therapeutic strategies aimed at elevating ASL pH, highlighting CFTR and cAMP modulation as a potential therapeutic strategy that could offer potential benefits to respiratory diseases characterized by ASL abnormalities, including CF and COPD.

## AUTHOR CONTRIBUTIONS

J.P.N. conducted apical fluid pH experiments, analyzed data, generated figures, performed the literature review, contributed to the manuscript conception, and drafted the manuscript. N.M. contributed to the manuscript conception and edited the manuscript. J.A.H. (Principal Investigator and Corresponding Author) provided oversight for the entire study, including data collection, analyses, manuscript drafting, and finalization.

## FUNDING INFORMATION

This research was supported by funding from the Canadian Institutes of Health Research (CIHR).

## CONFLICT OF INTEREST STATEMENT

The authors declare no conflicts of interest.

## Supporting information


**Figure S1.** Effect of PDE‐4 inhibitor Roflumilast and ABCC4 inhibitor MK‐571 post‐CFTR inhibition on apical fluid pH. Human airway epithelial (Calu‐3) cells were pre‐treated with CFTR inhibitors (a) CFTRinh‐172 (10 μM) and (b) GlyH‐101 (10 μM) to the apical (top) compartment for 30 min prior to treatment with PDE‐4 inhibitor roflumilast (1 μM) and ABCC4 inhibitor MK‐571 (10 μM) to the basolateral (bottom) compartment for 3 h. Measured apical fluid pH is depicted and comparisons between treatment groups were performed. Data presented as means ± SD (*n* = 4). A one‐way ANOVA with subsequent multiple comparisons was used for statistical analysis.

## Data Availability

All datasets generated and analyzed during this study are available from the corresponding author upon reasonable request.
