# Supplementary material for: Effects of cAMP and CFTR modulation on apical fluid pH in human airway Calu‐3 cells
Source: Physiol Rep. 2026 Feb 3;14(3):e70747. doi: 10.14814/phy2.70747 (PMC12868389; doi:10.14814/phy2.70747)
Supplement: Supplementary file 1 — Figure S1. Effect of PDE‐4 inhibitor Roflumilast and ABCC4 inhibitor MK‐571 post‐CFTR inhibition on apical fluid pH. Human airway epithelial (Calu‐3) cells were pre‐treated with CFTR inhibitors (a) CFTRinh‐172 (10 μM) and (b) GlyH‐101 (10 μM) to the apical (top) compartment for 30 min prior to treatment with PDE‐4 inhibitor roflumilast (1 μM) and ABCC4 inhibitor MK‐571 (10 μM) to the basolateral (bottom) compartment for 3 h. Measured apical fluid pH is depicted and comparisons between treatment groups were performed. Data presented as means ± SD (n = 4). A one‐way ANOVA with subsequent multiple comparisons was used for statistical analysis. [file PHY2-14-e70747-s001.docx]

**Supplementary Figures and Supplementary Figure Legends**

**Supplementary Figure 1:** **Effect of PDE-4 inhibitor Roflumilast and ABCC4 inhibitor MK-571 post-CFTR inhibition on apical fluid pH.** Human airway epithelial (Calu-3) cells were pre-treated with CFTR inhibitors (**a**) CFTRinh-172 (10 µM) and (**b**) GlyH-101 (10 µM) to the apical (top) compartment for 30 min prior to treatment with PDE-4 inhibitor roflumilast (1 µM) and ABCC4 inhibitor MK-571 (10 µM) to the basolateral (bottom) compartment for 3 h. Measured apical fluid pH is depicted and comparisons between treatment groups were performed. Data presented as means ± SD (n=4). A one-way ANOVA with subsequent multiple comparisons was used for statistical analysis.
